# Belowground Plant–Herbivore Interactions Vary among Climate-Driven Range-Expanding Plant Species with Different Degrees of Novel Chemistry

**DOI:** 10.3389/fpls.2017.01861

**Published:** 2017-10-25

**Authors:** Rutger A. Wilschut, Julio C. P. Silva, Paolina Garbeva, Wim H. van der Putten

**Affiliations:** ^1^Department of Terrestrial Ecology, Netherlands Institute of Ecology, Wageningen, Netherlands; ^2^Laboratory of Nematology, Wageningen University and Research, Wageningen, Netherlands; ^3^Department of Microbial Ecology, Netherlands Institute of Ecology, Wageningen, Netherlands

**Keywords:** range-expanding plant species, novel weapons, plant–herbivore interactions, root chemistry, root-feeding nematodes, volatile organic compounds, *Centaurea stoebe*

## Abstract

An increasing number of studies report plant range expansions to higher latitudes and altitudes in response to global warming. However, consequences for interactions with other species in the novel ranges are poorly understood. Here, we examine how range-expanding plant species interact with root-feeding nematodes from the new range. Root-feeding nematodes are ubiquitous belowground herbivores that may impact the structure and composition of natural vegetation. Because of their ecological novelty, we hypothesized that range-expanding plant species will be less suitable hosts for root-feeding nematodes than native congeneric plant species. In greenhouse and lab trials we compared nematode preference and performance of two root-feeding nematode species between range-expanding plant species and their congeneric natives. In order to understand differences in nematode preferences, we compared root volatile profiles of all range-expanders and congeneric natives. Nematode preferences and performances differed substantially among the pairs of range-expanders and natives. The range-expander that had the most unique volatile profile compared to its related native was unattractive and a poor host for nematodes. Other range-expanding plant species that differed less in root chemistry from native congeners, also differed less in nematode attraction and performance. We conclude that the three climate-driven range-expanding plant species studied varied considerably in their chemical novelty compared to their congeneric natives, and therefore affected native root-feeding nematodes in species-specific ways. Our data suggest that through variation in chemical novelty, range-expanding plant species may vary in their impacts on belowground herbivores in the new range.

## Introduction

One of the most evident ecological consequences of current climate change is the latitudinal and altitudinal range expansion of many plant and animal species (Walther et al., 2002; Parmesan, 2006; Le Roux and Mcgeoch, 2008). As not all species expand their range at similar rates (Berg et al., 2010), coevolved interactions between plants, aboveground and belowground organisms are likely to become disrupted, whereas novel interactions can be developed in the new range (Lavergne et al., 2010; Van Der Putten, 2012). Range-expanding plant species might benefit from these new biotic conditions when they do not encounter coevolved natural enemies in the expanded range (De Frenne et al., 2014; Dostálek et al., 2015). At the same time, range-expanders will become exposed to non-coevolved natural enemies that are native to these new areas. The strength of the enemy release effect will be largely determined by the inability of the novel natural enemies to exploit the range-expanders and the ability of the range-expanders to successfully defend themselves (Verhoeven et al., 2009). The present study was initiated in order to examine how root herbivores in the new range respond to range-expanding plant species.

Range-expanding plant species could benefit from the lack of coevolved novel natural enemies when they produce chemicals to which these enemies are not adapted. Such novel chemicals make the plants either less attractive or less digestible. For intercontinental introductions of exotic plant species, this possibility has been investigated under the “novel weapon hypothesis” (Callaway and Ridenour, 2004; Schaffner et al., 2011). Several studies have shown that invasive exotic plant species produce more unique shoot compounds than native plant species in the invaded range (Cappuccino and Arnason, 2006; Macel et al., 2014), thereby negatively affecting the performance of native aboveground invertebrate herbivores (Macel et al., 2014). The strength of novel weapon effects could differ between introduced exotic plant species and intra-continental range-expanders as more natural enemies may be shared between the original range and the new range of intra-continental range-expanders than of intercontinentally introduced exotic species. Yet, aboveground herbivores that lack a co-evolutionary history with both the range-expanding and the related native plant species performed less well on some successful range-expanders than on related natives (Engelkes et al., 2008). This suggests a role for plant chemistry in the success of range-expanding plants. However, the novel weapon hypothesis so far has not been tested in studies on intracontinental range-expanding plant species. Moreover, there is a paucity of studies testing the effects of novel chemistry on belowground herbivores, both for introduced exotics and intra-continental range-expanders.

In their new range, successful range-expanding plant species on average are less negatively affected by soil communities than congeneric natives (Van Grunsven et al., 2007; Engelkes et al., 2008). This effect has been explained by the on average lower accumulation of soil-borne fungal pathogens (Morriën and Van Der Putten, 2013) and root-feeding nematodes (Morriën et al., 2012) on the roots of range-expanding plant species than on congeneric natives. However, there is considerable variation in the outcome of plant-nematode interactions among range-expanding plant species (Morriën et al., 2012; Viketoft and Van Der Putten, 2014; Wilschut et al., 2016). A likely explanation for this variation that has not yet been studied is the role of novel plant chemistry. Therefore, the aim of the present study was to examine how differences in plant-nematode interactions between range-expanding and native plant species relate to differences in root chemistries. We compared preference and reproductive performance of root herbivores on range-expanders with congeneric plant species that are native in the new range, in order to confound our tests as minimal as possible with general differences in plant chemistry.

We tested the hypotheses that native generalist root-feeding nematodes (1) are more strongly attracted to native than to range-expanding plant species, (2) prefer native plant species over range-expanding plant species and (3) show higher reproduction on native than on range-expanding plant species. We studied differences in nematode attraction to single plants of all tested plant species (hypothesis 1), differences in nematode preference between range-expanders and related natives (hypothesis 2) and differences in nematode performance between range-expanders and related natives (hypothesis 3) under both lab and greenhouse conditions. As root volatiles are known to influence attraction of entomopathogenic nematodes (Rolfe et al., 2000; Rasmann et al., 2005; Turlings et al., 2012), we examined volatile profiles of all six plant species as they also may explain patterns in root-feeding nematode attraction and preference. Together, our results will contribute to the understanding of how novel chemistry might affect belowground plant–herbivore interactions of range-expanding plant species.

## Materials and Methods

### Plant Species and Seed Collections

We selected three plant species that recently expanded their range naturally from lower latitude areas to higher latitude areas in North–Western Europe and that have a related native species in their new range. Range-expanding plant species that were examined in the experiments were *Centaurea stoebe* L., *Geranium pyrenaicum* Burm. f., and *Rorippa austriaca* Crantz and their congeneric native species were *C. jacea* L., *G. molle* L., and *R. sylvestris* (L.) Besser. All six plant species now co-occur in riparian grassland areas in the eastern part of the Rhine-Waal area in The Netherlands. Therefore, these plant species are subjected to at least partly overlapping abiotic and biotic conditions. Range-expanding *R. austriaca* and *G. pyrenaicum* naturally established in the Netherlands at the end of the 19th century and are now widespread, while the first population of range-expanding *C. stoebe* in the Netherlands was recorded in the last decade of the 20th century (Floron, 2017). Seeds of all six plant species originate from natural areas in the Netherlands. Seeds of *C. stoebe*, *G. molle*, *R. austriaca*, and *R. sylvestris* were directly collected from single populations the field. Seeds of *C. jacea* were collected from mother plants that were grown in an outside experiment at NIOO-KNAW (Wageningen, The Netherlands) from seeds collected in a natural population. Seeds of *G. pyrenaicum* were delivered by the company Cruydthoeck (Nijeberkoop, Netherlands), that grows wild plants under field conditions from seeds that originate from natural field sites. For all experiments, seeds of *Centaurea* and *Geranium* species were surface-sterilized by washing for 3 min in a 10% bleach solution, followed by rinsing with demineralized water, after which they were germinated on glass beads. Due to their small size, seeds of both *Rorippa* species were not surface-sterilized, but directly germinated on sterilized soil. Seeds were germinated in a climate cabinet at 20/10°C and 16 h light/8 h darkness.

### Nematodes

We used cultures of two root-feeding nematode species, the ectoparasitic *Helicotylenchus pseudorobustus* Steiner (hereafter *Helicotylenchus*) and the sedentary endoparasitic *Meloidogyne hapla* Chitwood (hereafter *Meloidogyne*), originating from populations in The Netherlands. We selected these species as they both have a wide host range, are common and widely distributed throughout Europe (Bongers, 1988). Both used cultures were previously established in a greenhouse at NIOO-KNAW. The culture of *Helicotylenchus* on Marram grass (*Ammophila arenaria* L.) originates from nematodes collected from coastal dunes. The culture of *Meloidogyne* originates from nematodes collected from a field near Bovensmilde (Drenthe, Netherlands) which were subsequently cultured on tomato (*Solanum lycopersicum* L.).

### Nematode Choice Experiments

To study differences in nematode attraction and preference, we performed choice experiments on agar and in soil, where nematodes could move to one of two opposing treatments. To examine nematode *attraction* to a plant species, we planted one seedling of a species at one side and left the other side unplanted. To examine nematode *preference* for either natives or range-expanders we planted single seedlings of congeneric native and range-expanding plant species at opposing sides of the test units. As a control for attraction and preference, we examined nematode movement in test units without seedlings. We calculated the percentage of nematodes moving to either one of the sides of the test units.

#### Choice Experiment on Agar

To examine nematode choice *in vitro*, we used Petri dishes of 9 cm diameter filled with 20 ml 0.5% microbial agar (Merck kGaA, Germany) (Piskiewicz et al., 2009). We used eight independent replicates for each treatment. We placed 20-days-old seedlings 4 cm from the center of the Petri dish. Thereafter, the Petri dishes were placed in a climatized chamber at 16/8 h light/dark and 20°C. After 2 days, 20 μl of tap water suspension containing 40 juveniles of either *Helicotylenchus* or *Meloidogyne* was pipetted at the center of the Petri dishes. Nematode choice was examined 2 days after inoculation by counting using a stereo-microscope (200× magnification). We considered a nematode to be significantly attracted to one treatment when it moved at least 0.5 cm into the half of the Petri dish oriented toward that treatment.

#### Choice Experiment in Soil

To examine nematode choices under more natural conditions than on agar, we performed a choice experiment in soil-filled Y-tubes (Van Tol et al., 2001; Piskiewicz et al., 2009) in a greenhouse at 16/8 h light/dark and 20/15°C. We used six independent replicates for each treatment. Each Y-tube consisted of a core piece and two removable arms (see Supplementary Figure 1A), which were all filled with gamma-sterilized soil (25 KGray, Syngenta bv, Ede, Netherlands). The soil originated from a former agricultural field (Beneden-Leeuwen, Netherlands; N51° 53.952, E05° 33.670) in a riparian system where all plant species can occur. Prior to sterilization, the field soil was homogenized with sand at a rate of 2:1 (w:w) in order to reduce the relative clay content. Seedlings of 20 days old were planted in the Y-tube arms. Soil moisture was adjusted to 10% (w:w) and maintained at this level until nematode inoculation. Five days after planting the seedlings, 2 ml of water suspension with 200 *Helicotylenchus* or *Meloidogyne* juveniles was inoculated 2 cm deep in both sides of the core piece, to have an equal distribution of nematodes throughout the core piece. Then, both units with the planted seedlings were placed on the Y-tube and for the remaining experimental time the arms were moistened daily with 5 ml of demineralized water. After that, nematodes could enter an arm in which the roots were growing. Four days after inoculation, the two arms of the Y-tube were separated and nematodes from each arm and the core piece were extracted by Cobb’s decantation (Cobb, 1918) and counted using an inverted light microscope (200× magnification).

### Nematode Reproduction Experiment

For each plant species, ten 12-days-old seedlings were planted separately in 11 cm × 11 cm × 12 cm pots filled with soil homogenized and sterilized as explained above. The pots were placed in a greenhouse in a randomized block design with five replicate blocks. After 12 days, pots were inoculated with 2 ml water suspension with either 200 *Meloidogyne* or 200 *Helicotylenchus* juveniles. During the subsequent 16 weeks the pots were watered twice a week and kept on the same weight of approximately 870 g, of approximately 15% (w:w) soil moisture content. Thereafter, roots and soils were separated and used for nematode extraction. All roots were washed in 200 ml tap water, after which the washing water containing nematodes that were present in the rhizosphere was stored. Nematodes of each individual replicate were combined into a single sample by extracting all nematodes from the wash and soil using an Oostenbrink elutriator (Oostenbrink, 1960). Roots collected from pots inoculated with the ectoparasite *Helicotylenchus* were dried at 70°C. Roots from pots inoculated with *Meloidogyne* were split and both halves were weighed fresh. One half of the roots was dried at 70°C until constant weight, whereas the other half was cut into pieces of 1–2 cm and placed for 4 weeks in a mistifier to extract nematodes from the inside of the roots (Funnel-spray method; Oostenbrink, 1960). Total dry root biomass was assessed using total fresh root weight and fresh/dry weight ratio of each sample. Nematode suspensions were harvested from the mistifier after 2 and 4 weeks, combined, and concentrated to 10 ml. Nematodes were counted using an inverted light microscope (200× magnification).

### Root Volatile Analysis

To relate nematode attraction, preference, and performance to root chemistry, we analyzed root volatile profiles by Gas Chromatography Quadrupole Time of Flight (GC-QTOF) analysis.

#### Volatile Trapping

Four 20-days-old seedlings of each plant species were placed in individual 70 ml glass pots filled with sterilized soil (see choice experiment in soil). After 15 days, steel traps containing the volatile absorbants Tenax TA (150 mg) and Carbopack B (150 mg; Markes International Ltd., Llantrisant, United Kingdom) were attached at both sides of the glass pots (Supplementary Figure 1B). After 24 h of incubation the traps were removed, capped and stored at 4°C until GC-QTOF analysis.

#### GC-QTOF Analysis of Volatiles Compounds

The volatiles were collected from the traps using an automated thermos desorption unit (Unity TD-100, Markes International Ltd., Llantrisant, United Kingdom) at 210°C for 12 min (Helium flow 50 ml/min) and trapped on a cold trap at -10°C. The volatiles were introduced into the GC-QTOF (model Agilent 7890B GC and the Agilent 7200A QTOF, Santa Clara, CA, United States) by heating the cold trap for 3 min to 280°C. Split ratio was set to 1:10, and the column used was a 30 mm × 0.25 mm ID RXI-5MS, film thickness 0.25 μm (Restek 13424-6850, Bellefonte, PA, United States). The following temperature program was used: 39°C for 2 min, from 39 to 95°C at 3.5°C/min, then to 165°C at 6°C/min, to 250°C at 15°C/min and finally to 300°C at 40°C/min and 20 min at 300°C. The volatiles were detected by a mass spectrometer (MS) operating at 70 eV in EI mode. Mass spectra were acquired in full-scan mode (30–400AMU, 4 scans/s). GC-MS-data were collected and converted to a mzData file using the Chemstation B.06.00 (Agilent Technologies, United States). Data were further processed with MZmine 2.14.2 (Pluskal et al., 2010) with the tools mass detection (centroid mode, noise level = 1000), chromatogram builder (min time span = 0.05 min, min height = 1.5E03, m/z tolerance of 1 m/z or 5 ppm), and chromatogram deconvolution (local minimum search, chromatographic threshold = 40%, Min in RT range = 0.1 min, Min relative height = 2.0%, Min absolute height = 1.5E03, Min ratio of peak top/edge = 2, peak duration = 0.0–0.5 min). Detected and deconvoluted peaks were identified by their mass spectra using NIST MS Search and NIST 2014 (National Institute of Standards and Technology, United States) and aligned using Random Sample Consensus (RANSAC) aligner (mz tolerance = 1 m/z or 5 ppm, RT tolerance = 0.1, RT tolerance after correction = 0.05, RANSAC iteration = 10000, Min number of points = 60%, threshold value = 0.1). Processed data were exported for further statistical analysis as explained under ‘Statistical analysis.’ The identification of detected compounds was further evaluated using the software AMDIS 2.72^1^ (Stein, 1999). The retention indexes were calculated for each compound and compared with those found in NIST 2014 and in-house databases.

### Statistical Analyses

Differences in nematode attraction and preference were tested by pair-wise *t*-tests in SigmaPlot (Systat software, Inc.). Overall differences in nematode attraction between natives and range-expanders were tested using general linear models with origin as fixed factor and plant species as random factor (packages lme4 and lmerTest; Bates et al., 2014; Kuznetsova et al., 2015) using R studio (version 0.98.507; R Core Development Team, 2012). Differences in nematode numbers between plant species were tested for each nematode species separately using generalized linear models with a negative binomial error distribution (MASS package; Venables and Ripley, 2013) modeling fixed factors ‘plant species’ and ‘experimental block’. Wald *post hoc* tests were then used to test for differences between plant species using the phia package (De Rosario-Martinez, 2013). Using Pearson correlation tests, we examined whether nematode reproduction corresponded with nematode attraction in the y-tubes. Analyses on volatile data were performed using MetaboAnalyst V3.0^2^ (Xia et al., 2015). Prior to One-way ANOVA and multivariate analyses (PLS-DA) data were normalized via log-transformation and auto scaling. To identify mass features significantly differing between plant species, a one-way-ANOVA with *post hoc* Tukey HSD-tests was performed. Mass features were considered to be statistically relevant when *p*- and FDR-values were ≤ 0.05.

## Results

### Nematode Attraction

**FIGURE 1:**
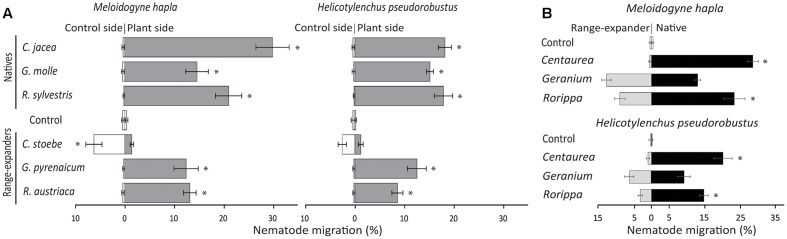
**(A)** Attraction or repellence (% individuals migrated) of the nematode species *Meloidogyne hapla* and *Helicotylenchus pseudorobustus* by native and range-expanding plant species in sterilized soil. **(B)** Nematode preference between native plant species *Centaurea jacea*, *Geranium molle*, and *Rorippa sylvestris* and congeneric range-expanders *C. stoebe*, *G. pyrenaicum*, and *R. austriaca*. In both panels horizontal bars show averages ± standard errors and asterisks represent significant paired *t*-test values (*p* < 0.05) between empty control and plant **(A)** or between native and range-expanding plant species **(B)**.

First, we confirmed that the controls in the nematode attraction experiments were effective. Indeed, when the tests were performed in the absence of plants both on agar and in soil neither *Helicotylenchus* nor *Meloidogyne* showed significant movement away from the point of addition (**Figure 1** and Supplementary Figure 2).

#### Meloidogyne

On average, there was a trend of stronger attraction of *Meloidogyne* to natives than to range-expanding plant species on agar (natives: 25.3 ± 3.6%, range-expanders: 10.9 ± 3.9%; *F* = 7.56, *p* = 0.051), but this was not significant in soil (natives: 21.9 ± 4.4%, range-expanders: 9.0 ± 3.8%; *F* = 4.86, *p* = 0.09). On agar, all natives significantly attracted *Meloidogyne* away from the empty control (all *t*-values > 3.48, all *p*-values < 0.05; Supplementary Figure 2A), whereas none of the range-expanders did so (Supplementary Figure 2A). In soil, all three native species significantly attracted *Meloidogyne* away from the empty controls (all *t*-values > 6.65, all *p*-values < 0.01; **Figure 1A**). Both range-expanding *Geranium* and *Rorippa* also attracted *Meloidogyne* away from the empty control in soil (*t*-values > 4.84, *p*-values < 0.01; **Figure 1A**). Interestingly, the range-expanding *Centaurea* significantly repelled *Meloidogyne* toward the empty control in both agar and soil (*t*-values < -3.21, *p*-values < 0.05; **Figure 1A** and Supplementary Figure 2A). Thus, all natives significantly attracted *Meloidogyne*, whereas range-expanders either repelled *Meloidogyne* or attracted *Meloidogyne* only in one of the two test units.

#### Helicotylenchus

On average, native plant species did not attract *Helicotylenchus* more strongly than range-expanders on agar (natives: 21.9 ± 8.0%, range-expanders: 13.6 ± 2.4%; *F* = 0.99, *p* = 0.38), while they did so in soil (natives: 17.2 ± 0.8%, range-expanders: 7.4 ± 3.3%; *F* = 7.83, *p* < 0.05). Individually, all native plant species significantly attracted *Helicotylenchus* in both test units, when compared to empty controls (all *t*-values > 3.2, all *p*-values < 0.05; **Figure 1A** and Supplementary Figure 2A). On agar only range-expanding *Geranium* significantly attracted *Helicotylenchus* away from the empty control (*t* = 4.34, *p* < 0.01; Supplementary Figure 2A), while in soil both range-expanding *Geranium* and *Rorippa* did so (*t*-values > 6.57, *p*-values < 0.01; **Figure 1A**). Range-expanding *Centaurea* significantly repelled *Helicotylenchus* toward the empty control on agar (*t* = -2.83, *p* < 0.05; Supplementary Figure 2A), but not in soil (*t* = -1.98, *p* = 0.10; **Figure 1A**). Overall, native plant species always significantly attracted *Helicotylenchus*, whereas attraction and repellence by range-expanding plant were species-specific and depended on test unit.

### Nematode Preference

*Meloidogyne* and *Helicotylenchus* preferred native *Centaurea* and *Rorippa* over their congeneric range-expanders (*t*-values > 3.68, *p*-values < 0.05; **Figure 1B** and Supplementary Figure 2B), although the preference of *Helicotylenchus* for native *Rorippa* was not significant on agar (*t* = 1.47, *p* = 0.19). Both *Meloidogyne* and *Helicotylenchus* did not show a preference for either native or range-expanding *Geranium* on either agar or in soil (all *t*-values < 1.59, all *p*-values > 0.15; **Figure 1B** and Supplementary Figure 2B). Therefore, our results show that two out of three native plant species were preferred over related range-expanding plant species by both nematode species, whereas in the third plant pair both nematode species did not show a preference for either the native or the range-expander.

### Nematode Reproductive Performance

**FIGURE 2:**
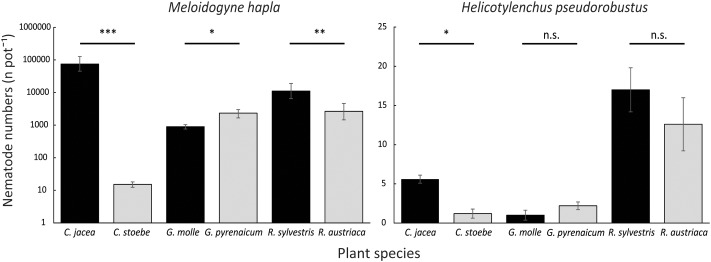
Mean total numbers (N pot^-1^) of root-feeding nematodes *M. hapla* (left; logarithmic scale) and *H. pseudorobustus* (right; linear scale) on range-expanding plant species *C. stoebe*, *G. pyrenaicum*, and *R. austriaca* (gray), and congeneric natives *C. jacea*, *G. molle*, and *R. sylvestris* (black). Vertical bars show means ± standard errors. Asterisks indicate levels of significance (^∗^*p* < 0.05, ^∗∗^*p* < 0.01, ^∗∗∗^*p* < 0.001, n.s., not significant) of pairwise *post hoc* Wald tests within plant pairs.

**FIGURE 3:**
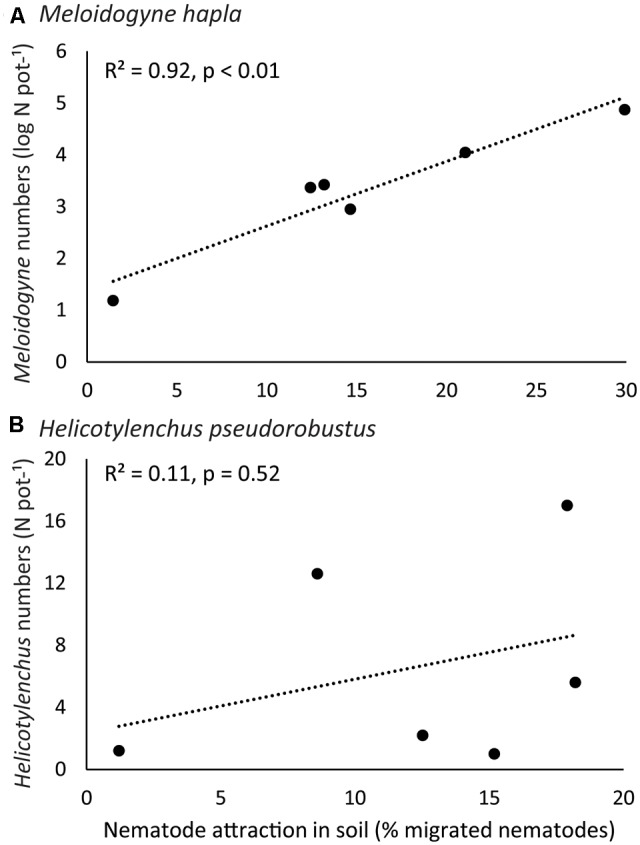
Correlation plots between nematode attraction (*x*-axis) and nematode reproduction (*y*-axis), for root-feeding nematodes **(A)** *Meloidogyne hapla* and **(B)** *H. pseudorobustus*. Dots represent the six different plant species tested. *R*^2^-values and *p*-values of the Pearson correlation tests are given.

*Meloidogyne* reproduction differed significantly among plant species (explained deviance = 182.45, *p* < 0.0001). *Meloidogyne* numbers were higher on native *C. jacea* than on range-expanding *C. stoebe* (χ^2^ = 251.94, *p* < 0.0001; **Figure 2**) and higher on native *R. sylvestris* than on range-expanding *R. austriaca* (χ^2^ = 12.18, *p* < 0.001; **Figure 2**). However, in *Geranium*, *Meloidogyne* numbers were higher on the range-expander *G. pyrenaicum* than on the native *G. molle* (χ^2^ = 5.87, *p* < 0.05; **Figure 2**). *Helicotylenchus* numbers also differed significantly among plant species (explained deviance = 114.05, *p* < 0.0001; **Figure 2**). There were significantly more *Helicotylenchus* on native *C. jacea* than on range-expander *C. stoebe* (χ^2^ = 10.10, *p* < 0.05; **Figure 2**). However, *post hoc* analysis of the other two plant pairs did not reveal any significant differences in *Helicotylenchus* numbers between range-expanders and congeneric natives. *Meloidogyne* numbers per plant species strongly correlated with the attraction by these plant species in y-tubes (*R*^2^ = 0.92, *p* < 0.01; **Figure 3A**), while this correlation was not significant for *Helicotylenchus* (*R*^2^ = 0.11, *p* = 0.52; **Figure 3B**).

### Root Volatiles

**FIGURE 4:**
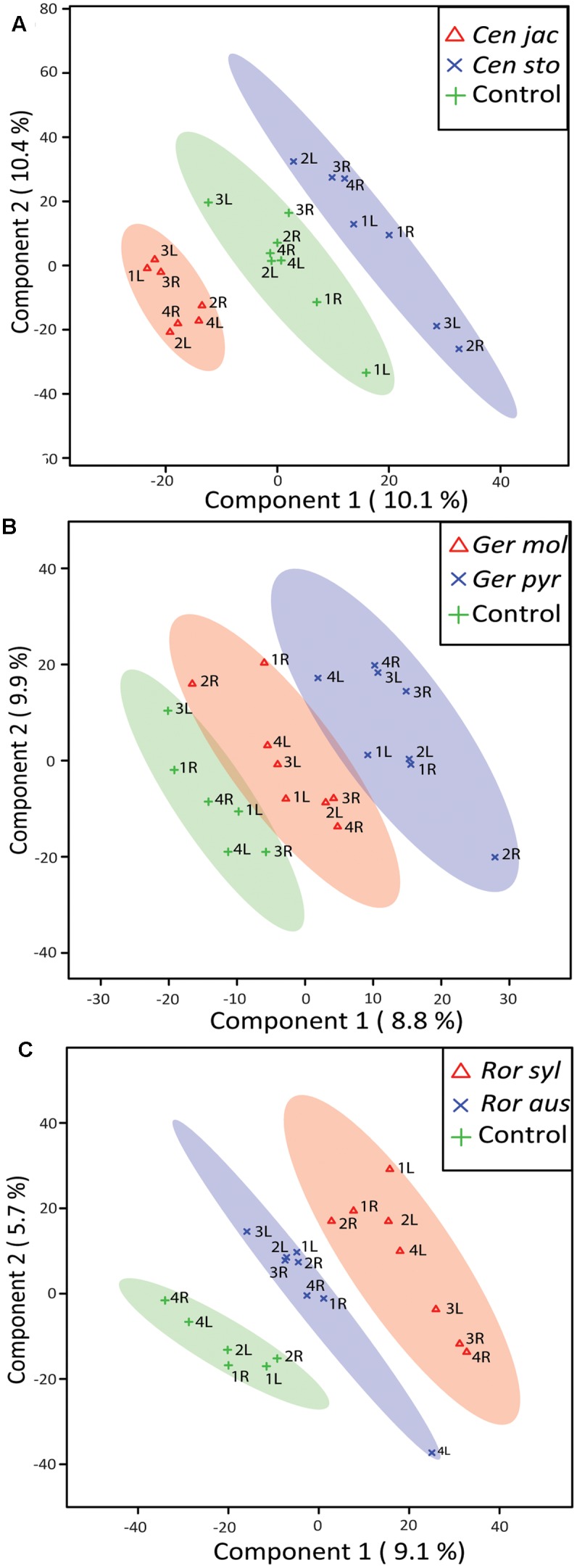
Partial least square-discriminant analysis (PLS-DA) score plots of root volatile profiles measured with GC-QTOF-MS. The semi-transparent ovals outline the 95% confidence intervals of natives (red triangles), range-expanders (blue crosses) and sterilized control soils (green crosses) for *Centaurea* **(A)**, *Geranium* **(B)**, and *Rorippa* **(C)**. Sample numbers and position of the volatile trap (left or right) are given.

We detected 1964 putative volatile compounds in all samples, of which approximately 25% (491 volatile compounds) were produced by plants (Supplementary Figure 3). The other 1473 volatile compounds were detected in the tubes containing only gamma-sterilized soil. When the root volatiles of all six plant species were analyzed together, the strongest overlap between species was found within the pairs of congeneric species, indicating that chemistry varies more strongly between genera than within genera (Supplementary Figure 4). Within the *Centaurea* pair 21 volatile compounds were significantly different between the native and range-expander, resulting in a clear separation of their volatile profiles (**Figure 4A**). Five of these compounds were detected only in the headspace of *C. stoebe*: indene, tridecane and nonadecane (alkanes), 1,2-benzisothiazole (benzenoids/ketone) and alpha-gurjunene (sesquiterpene), and three volatiles were detected only in the headspace of the native *C. jacea*: petasitene (sesquiterpene), benzophenone (benzenoids/ketone), and an unknown terpene (**Table 1**). Thirteen compounds where found in both *Centaurea* species, but in different abundances (**Table 1**). Volatile profiles from native and range-expanding *Geranium* and *Rorippa* were less clearly separated in the PLS-DA score plots, although samples from controls, native and range-expanding plants could still be divided into three distinct groups with 95% confidence intervals (**Figures 4B,C**). There were 11 volatiles that showed significant differences between the *Geranium* species and 6 between the *Rorippa* species (all *p*-values < 0.05). Native *G. molle* produced five unique volatile compounds, compared to four by range-expanding *G. pyrenaicum*, while two volatiles differed in production levels between the species. The native *R. sylvestris* produced four unique compounds compared to two unique compounds that were produced exclusively by the range-expander *R. austriaca*. Therefore, differences in volatile profiles between range-expanders and congeneric natives depended on the species pair; in two out of three cases, the range-expander produced fewer unique volatiles than the congeneric native.

**Table 1:** Volatile organic compounds produced by native *Centaurea jacea* and range-expanding *C. stoebe*.

Compound name	RT	ELRI	Plant
Sulfur dioxide	2.04	488	CJ
Dimethylsulfide	2.4	529	CS
Carbon disulfide	2.5	541	CJ
Furan, 2-methyl	2.9	583	CJ
1,3-dioxolane, 2-methyl-	3.4	639	CS
benzene 1,2 dimethyl	10.1	890	CJ
Dimethyl sulfone	10.9	916	CS
Dimethyl trisulfide	13.1	963	CS
Mesitylene	14.3	990	CJ
Indene^∗∗^	15.7	1023	CS
Acetophenone	17.4	1062	CS
1,2-benzisothiazol^∗∗^	23.9	1229	CS
Tridecane^∗∗^	26.8	1299	CS
Petasitene^∗^	30.1	1398	CJ
Alpha-gurjunene^∗∗^	30.4	1407	CS
Unknown terpene^∗^	32.73	1448	CJ
Phenyl maleic anhydride	34.29	1534	CJ
Benzophenone^∗^	36.9	1620	CJ
Pentadecanoic acid	40.02	1867	CS
Nonadecane^∗∗^	40.4	1901	CS
Diphenylsulfone	40.7	1934	CS


*Tentative compound names are shown, which are based on retention time (RT) and ELRI (Experimental linear retention index) values, measured with GC-QTOF-MS. All compounds are significantly more produced by either *C. jacea* (CJ) or *C. stoebe* (CS). Compounds that are produced solely by *C. jacea* are indicated with ‘^∗^’ and compounds produced solely by *C. stoebe* with ‘^∗∗^.’*

## Discussion

Several studies have proposed that invasiveness of intercontinentally introduced exotic plant species can be enhanced by their novel chemistry, e.g., through allelopathy (Callaway and Aschehoug, 2000; Zheng et al., 2015), or by the suppression of the local natural enemies (Schaffner et al., 2011). Yet, little is known about the effects of novel chemistry of intra-continental climate-driven range-expanders on communities in the new range. Moreover, empirical studies testing novel chemistry effects on belowground plant–herbivore interactions in the novel range are lacking. Here, we show that root-feeding nematodes from the novel range were strongly attracted to native plant species, while, in support of our hypothesis, the average attraction by range-expanders mostly was less strong. Yet, we also found substantial differences in nematode attraction among range-expanding plant species: while the range-expanding *C. stoebe* repelled both nematode species in at least one of the attraction experiments, range-expanding *G. pyrenaicum* and *R. austriaca* attracted nematodes. Therefore, we show that some range-expanding plant species will attract considerable amounts of root-feeding nematodes in their new range, while other species will repel them, potentially leading to profound differences in herbivore pressure between range-expanders in their new range.

In test units with both natives and congeneric range-expanders, both nematode species preferred native *Centaurea* and *Rorippa* over their congeneric range-expanders, while our hypothesis of stronger nematode preference for natives was not confirmed when comparing the *Geranium* species. In plant communities in the new range, the preference for native plant species could lead to apparent competition (Holt, 1977), when natives experience stronger herbivore pressure (Orrock et al., 2008), leading to indirect competitive benefits for the range-expanders. For *Meloidogyne*, reproduction strongly corresponded with the attraction to the different plant species, as we found that *Meloidogyne* reproduction was significantly higher in the roots of native *Centaurea* and *Rorippa* than in the roots of their congeneric range-expanders. Notably, the differences in *Meloidogyne* reproduction between the *Centaurea* species were more substantial than between the *Rorippa* species. This was especially due to poor nematode reproduction on the range-expanding *C. stoebe*, which is in line with a previous study (Wilschut et al., 2016). *Helicotylenchus* numbers did not fully correspond with the attraction to the different plant species. Although they were lower in the rhizosphere of range-expanding *Centaurea* than in that of native *Centaura*, no differences were found in the other two plant pairs. The overall very low *Helicotylenchus* numbers indicate that no – or hardly any – reproduction of this species took place in this experiment. While the species did show profound chemical attraction to some of the plant species, we could therefore not properly estimate differences in performance on these different plant species.

Contrary to our hypothesis, but in line with a previous study (Wilschut et al., 2016), the range-expanding *Geranium* hosted slightly higher numbers of *Meloidogyne* than the native *Geranium*, indicating that not all range-expanding plant species are poorer nematode hosts than congeneric natives. Depending on naivety of either the host plant species or the herbivore in a novel plant–herbivore novel interaction, herbivore performance can be found to be strong or weak (Verhoeven et al., 2009). We did not perform experiments using *Meloidogyne* and *Helicotylenchus* populations from the original range of the range-expanding plant species, so our data do not allow to draw conclusions on nematode preference and performance of the range-expanding plant species in their native range. However, as gene flow between soil-born nematode populations is expected to be low (Blouin et al., 1999), a certain degree of local adaptation is well possible, so that it may well be that the nematode populations in the new range differ, at least to some extent, from populations in the original range. The use of nematode populations originating from natural areas in the new range and the subsequent culturing on plant species that is phylogenetically unrelated to the examined plant species allowed a phylogenetically unbiased test of the effects of the natural co-evolutionary histories between the nematode and plant species on nematode attraction and performance.

We expected that the patterns in nematode attraction, preference and reproduction found in the present study would be caused by differences in root chemistry between native and range-expanding plant species. Indeed, the analyses of volatile compounds revealed that range-expanding *C. stoebe* produced more unique volatile compounds than native *C. jacea*. These results correspond with a study on aboveground herbivores, in which herbivore performance was also shown to be low on range-expanding and exotic plants with more unique chemistry than their related natives (Macel et al., 2014). In addition to higher numbers of unique compounds, our study also reveals differences in the production levels of several shared volatile compounds between the *Centaurea* species. Therefore, the nematode repellence and the poor nematode reproduction on the range-expanding *C. stoebe*, compared to the native *C. jacea*, might be explained by both the production of higher numbers of unique compounds and by different production levels of shared compounds. Interestingly, novel chemistry of *C. stoebe* has also been related to the poor performance of aboveground generalist herbivores in North America (Schaffner et al., 2011), where this plant species is invasive. In contrast to range-expanding *Centaurea*, both range-expanding *Rorippa* and *Geranium* produced fewer unique volatiles than their congeneric natives. Differences in volatile profiles were stronger in *Geranium* than in *Rorippa*, which was not reflected in the patterns of nematode preference and reproduction. Native *Rorippa* hosted higher nematode numbers and was more attractive to both nematode species than range-expanding *Rorippa*, while in *Geranium* there was no clear nematode preference for either the native or the range-expander, and nematode reproduction levels were higher in the range-expander than in the native. These results suggest that when unique volatile compounds play a role in nematode attraction or distraction, the identity, rather than the number of unique compounds may influence the outcome of plant-nematode interactions. Interestingly, but not unexpectedly, the differences in volatile profiles between all three pairs of congeneric native and range-expanding plant species were smaller than the differences among the three genera. This suggests that while root-feeding nematode species such as *Meloidogyne* have adapted to plant species with strongly different root chemistries, they may still perform poorly on range-expanding plant species that possess root chemistries slightly deviating from that of the plant species the nematodes are adapted to.

Our volatile analyses revealed, next to many plant volatiles, a large diversity of volatiles emitted by gamma-sterilized soils, which is in line with earlier studies (Schulz-Bohm et al., 2015; Kai et al., 2016). Possibly, the chemical background of the soil caused the differences in nematode attraction between the tests on agar and soil, namely the higher numbers of nematodes moving to the unplanted side on agar. Alternatively, this effect could be caused by a stronger diffusion of root metabolites in the Petri dishes than in the soil-filled Y-tubes, resulting in a more equal distribution of root metabolites throughout the Petri dishes. Based on the differences between the two choice experiments we therefore conclude that choice experiments with root-feeding nematodes should preferably be performed in soil.

The application of GC-QTOF for volatile analysis allowed to obtain the tentative identification of the measured root volatiles. We identified several volatile compounds that were only detected in range-expanding *C. stoebe*, and therefore could cause the nematode-repelling effect found for this plant species. Root-emitted volatiles are known to play versatile roles in long distance below-ground interactions (Erb et al., 2013; Van Dam and Bouwmeester, 2016) and some of the volatile compounds identified in the present study have been shown to negatively affect nematodes (Piluk et al., 1998). Future studies testing the identified metabolites in different combinations and ratios could reveal which compounds cause the nematode-repelling effect found in *C. stoebe*. Yet, pin-pointing of the observed effects to a single volatile compound can be complicated, because nematodes might react to a blend of volatiles, rather than to single compounds (Mccormick et al., 2012).

Successful range-expanding plant species have been shown to be better defended against naïve aboveground generalist herbivores than congeneric native plant species (Engelkes et al., 2008), indicating that they may possess superior defense mechanisms compared to related native species in the new range. Such defense mechanisms may especially be effective when they are novel to the natural enemies in the new range. Our results suggest that together with the release of soil enemies from the original range (Van Grunsven et al., 2007), the possession of novel chemistry could explain why range-expanding plant species are less negatively affected by soil communities than related native plant species (Van Grunsven et al., 2007; Engelkes et al., 2008). As range-expanding plant species without closely related species in the new range are likely to possess the most unique root chemistries compared to native species present in the community, a phylogenetic approach (as in Strauss et al., 2006) may be considered to forecast which range-expanding plant species have the strongest potential to affect native communities in their novel range (Gilbert and Parker, 2016).

## Conclusion

We provide evidence that novel belowground chemistry of the root system of range-expanding plant species may suppress root herbivores in the new range. A range-expander that had the most different root chemistry compared to its related native suppressed root-feeding nematodes more strongly than range-expanders with root chemistries that were more comparable to those of related natives. However, our study included six plant species from three genera. Therefore, while our results elucidate the variation in potential impact of range-expanding plant species on native communities in their novel range, further studies are needed in order to be able to generalize these results and predict which range-expanding plant species may have strong impacts on native communities in the future.

## Author Contributions

All authors contributed to the design of the study. Greenhouse experiments were performed by JS and RW. The volatile experiment was performed by PG and JS. Data analyses were performed by RW, PG, and JS. The manuscript was written by RW with the help of all other authors.

## Conflict of Interest Statement

The authors declare that the research was conducted in the absence of any commercial or financial relationships that could be construed as a potential conflict of interest.

## Footnotes

1 http://chemdata.nist.gov/

2 www.metaboanalyst.ca
