# Supplementary material for: Belowground Plant–Herbivore Interactions Vary among Climate-Driven Range-Expanding Plant Species with Different Degrees of Novel Chemistry
Source: Front Plant Sci. 2017 Oct 25;8:1861. doi: 10.3389/fpls.2017.01861 (PMC5660973; doi:10.3389/fpls.2017.01861)
Supplement: Supplementary file 1 [file Data_Sheet_1.DOCX]

Supplementary Material

**Belowground plant-herbivore interactions vary among climate-driven range-expanding plant species with different degrees of novel chemistry**

Rutger A. Wilschut, Julio Carlos Pereira da Silva, Paolina Garbeva, Wim H. van der Putten

**Correspondence:** Rutger Wilschut: [r.wilschut@nioo.knaw.nl](mailto:r.wilschut@nioo.knaw.nl)


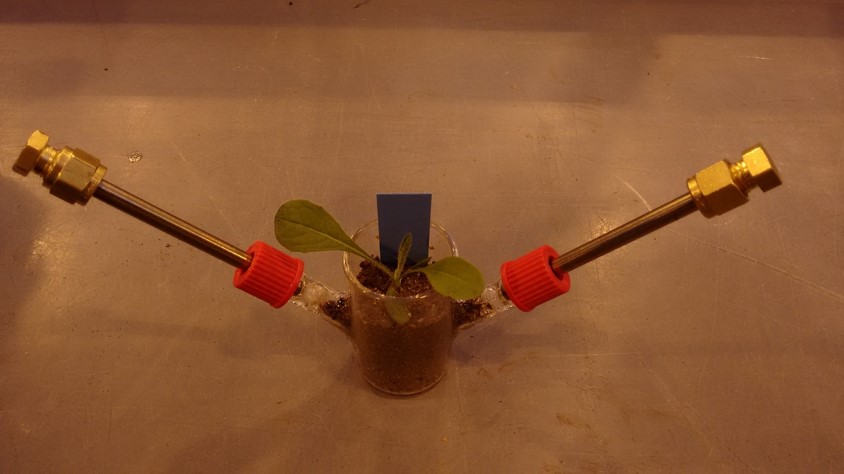

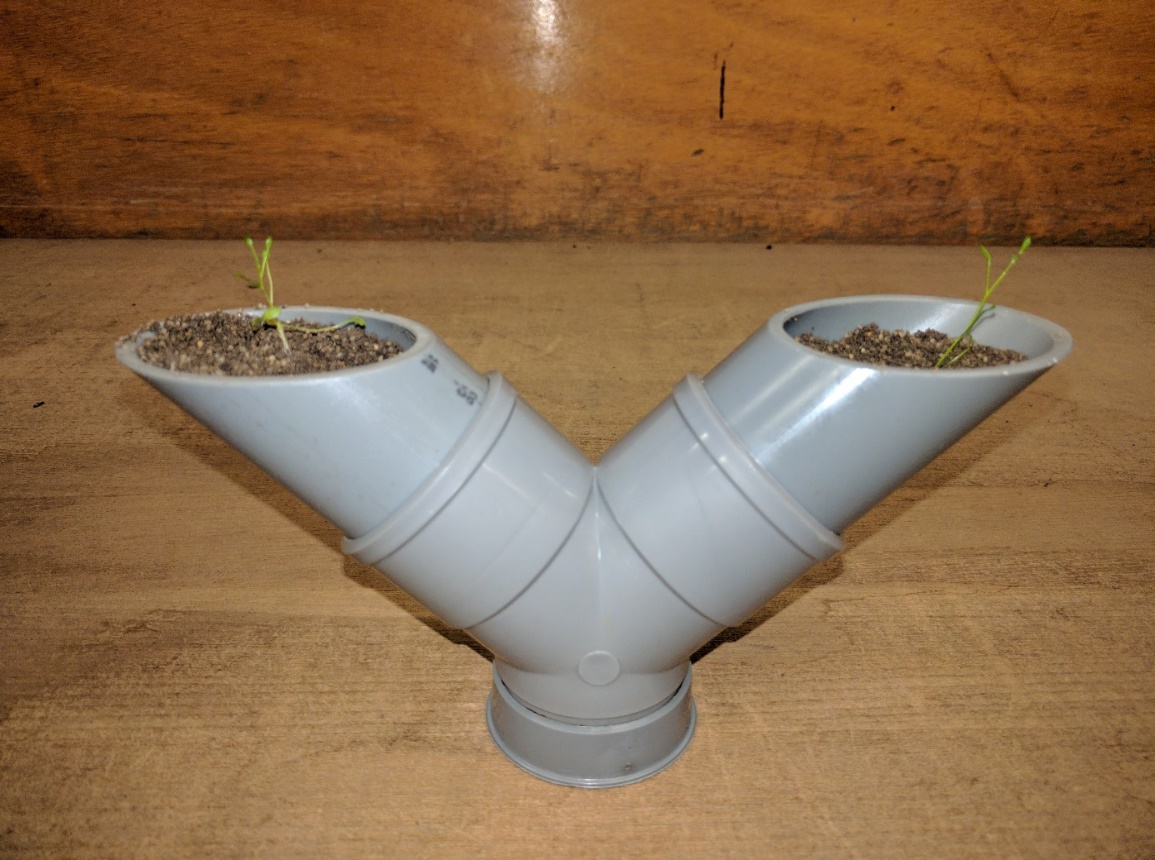


(A)

(B)

**Supplementary Figure 1.** Soil-filled y-tube used for nematode attraction and preference experiments (A) and volatile trap set-up containing two volatile traps attached to a soil-filled glass jar (B).
