# Supplementary material for: Belowground Plant–Herbivore Interactions Vary among Climate-Driven Range-Expanding Plant Species with Different Degrees of Novel Chemistry
Source: Front Plant Sci. 2017 Oct 25;8:1861. doi: 10.3389/fpls.2017.01861 (PMC5660973; doi:10.3389/fpls.2017.01861)
Supplement: Supplementary file 2 [file Data_Sheet_2.DOCX]

Supplementary Material

**Belowground plant-herbivore interactions vary among climate-driven range-expanding plant species with different degrees of novel chemistry**

Rutger A. Wilschut, Julio Carlos Pereira da Silva, Paolina Garbeva, Wim H. van der Putten

**Correspondence:** Rutger Wilschut: [r.wilschut@nioo.knaw.nl](mailto:r.wilschut@nioo.knaw.nl)


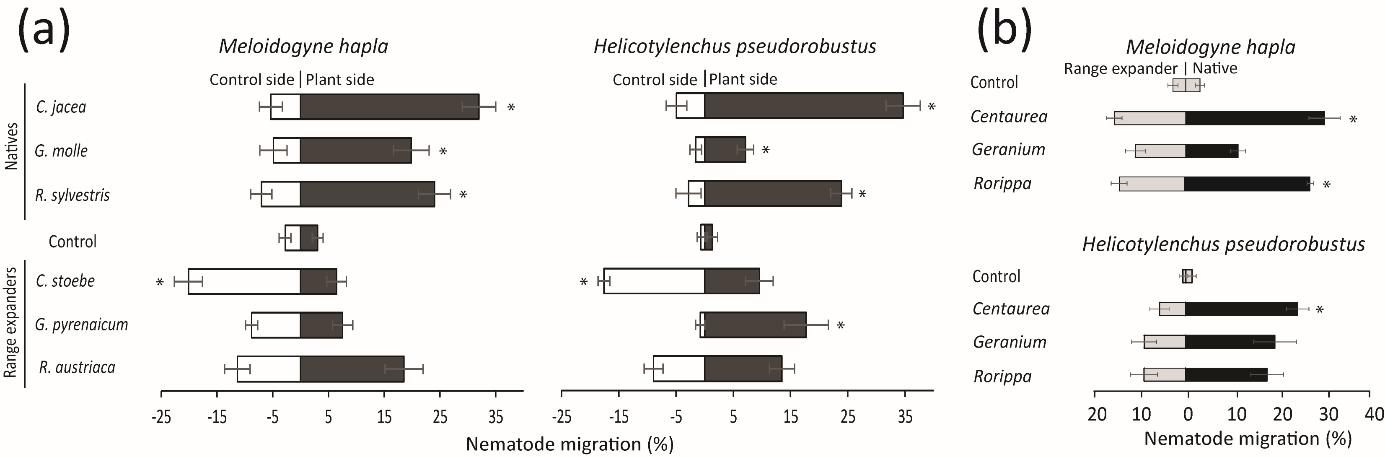


**Supplementary Figure 2.**  (A) Attraction or repellence (% individuals migrated) of the nematode species *Meloidogyne hapla* and *Helicotylenchus pseudorobustus* by native and range-expanding plant species on agar. Grey bars represent the plant sides and white bars the control sides of the agar plates. (B) Nematode choice between native plant species *Centaurea jacea*, *Geranium molle* and *Rorippa sylvestris* (dark grey) and congeneric range-expanders *Centaurea stoebe*, *Geranium pyrenaicum* and *Rorippa austriaca* (light grey). In both panels horizontal bars show averages ± standard errors and asterisks represent significant paired t-test values (p < 0.05) between empty control and plant (a) or between native and range-expanding plant species (B).
