# Supplementary material for: Belowground Plant–Herbivore Interactions Vary among Climate-Driven Range-Expanding Plant Species with Different Degrees of Novel Chemistry
Source: Front Plant Sci. 2017 Oct 25;8:1861. doi: 10.3389/fpls.2017.01861 (PMC5660973; doi:10.3389/fpls.2017.01861)
Supplement: Supplementary file 3 [file Data_Sheet_3.DOCX]

Supplementary Material

**Belowground plant-herbivore interactions vary among climate-driven range-expanding plant species with different degrees of novel chemistry**

Rutger A. Wilschut, Julio Carlos Pereira da Silva, Paolina Garbeva, Wim H. van der Putten

**Correspondence:** Rutger Wilschut: [r.wilschut@nioo.knaw.nl](mailto:r.wilschut@nioo.knaw.nl)

**Supplementary Figure 3.**  Total volatile numbers found in pots grown with range-expanding plant species Centaurea stoebe (C.s), Geranium pyrenaicum (G.p) and Rorippa austriaca (R.a) (black) and related native species Centaurea jacea (C.j), Geranium molle (G.m) and Rorippa sylvestris (R.s) (grey). Vertical bars show averages ± standard errors.
