# Supplementary material for: Belowground Plant–Herbivore Interactions Vary among Climate-Driven Range-Expanding Plant Species with Different Degrees of Novel Chemistry
Source: Front Plant Sci. 2017 Oct 25;8:1861. doi: 10.3389/fpls.2017.01861 (PMC5660973; doi:10.3389/fpls.2017.01861)
Supplement: Supplementary file 4 [file Data_Sheet_4.DOCX]

Supplementary Material

**Belowground plant-herbivore interactions vary among climate-driven range-expanding plant species with different degrees of novel chemistry**

Rutger A. Wilschut, Julio Carlos Pereira da Silva, Paolina Garbeva, Wim H. van der Putten

**Correspondence:** Rutger Wilschut: [r.wilschut@nioo.knaw.nl](mailto:r.wilschut@nioo.knaw.nl)


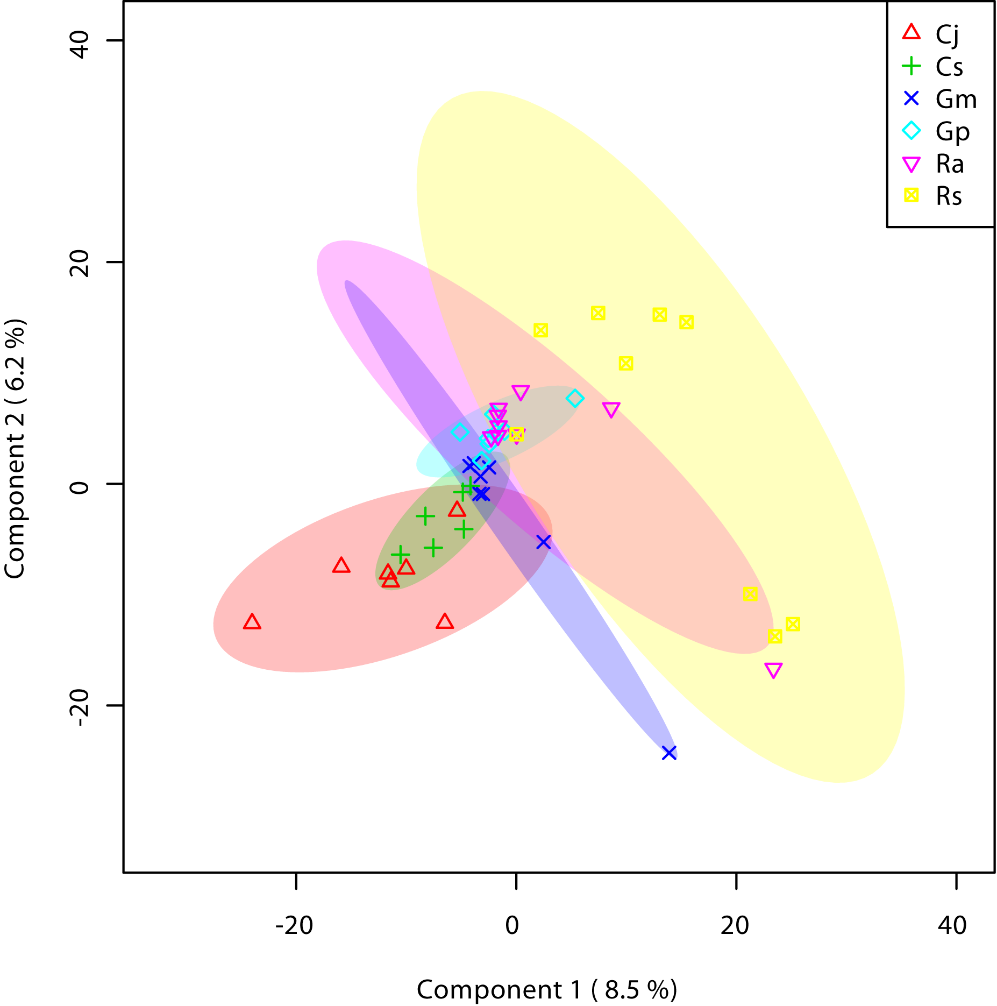


**Supplementary Figure 4.**  Partial least square-discriminant analysis (PLS-DA) score plots of root volatile profiles measured with GC-QTOF-MS. The semi-transparent ovals outline the 95% confidence intervals of native plant species *Centaurea jacea* (Cj), *Geranium molle* (Gm) and *Rorippa sylvestris* (Rs), and congeneric range-expanding plant species *Centaurea stoebe* (Cs), *Geranium pyrenaicum* (Gp) and *Rorippa austriaca* (Ra).
